# Genomic data provide new insights on the demographic history and the extent of recent material transfers in Norway spruce

**DOI:** 10.1111/eva.12801

**Published:** 2019-04-30

**Authors:** Jun Chen, Lili Li, Pascal Milesi, Gunnar Jansson, Mats Berlin, Bo Karlsson, Jelena Aleksic, Giovanni G. Vendramin, Martin Lascoux

**Affiliations:** ^1^ Department of Ecology and Genetics, Evolutionary Biology Centre Uppsala University Uppsala Sweden; ^2^ Forestry Research Institute of Sweden (Skogforsk) Uppsala Sweden; ^3^ Forestry Research Institute of Sweden (Skogforsk) Ekebo Sweden; ^4^ Institute of Molecular Genetics and Genetic Engineering University of Belgrade Belgrade Serbia; ^5^ Division of Florence, Institute of Biosciences and BioResources National Research Council (IBBR‐CNR) Sesto Fiorentino Italy

**Keywords:** demographic inferences, forest management, *Picea abies*, population transfer

## Abstract

Primeval forests are today exceedingly rare in Europe, and transfer of forest reproductive material for afforestation and improvement has been very common, especially over the last two centuries. This can be a serious impediment when inferring past population movements in response to past climate changes such as the last glacial maximum (LGM), some 18,000 years ago. In the present study, we genotyped 1,672 individuals from three *Picea* species (*P. abies*, *P. obovata*, and *P. omorika*) at 400K SNPs using exome capture to infer the past demographic history of Norway spruce (*P. abies*) and estimate the amount of recent introduction used to establish the Norway spruce breeding program in southern Sweden. Most of these trees belong to *P. abies* and originate from the base populations of the Swedish breeding program. Others originate from populations across the natural ranges of the three species. Of the 1,499 individuals stemming from the breeding program, a large proportion corresponds to recent introductions from mainland Europe. The split of *P. omorika* occurred 23 million years ago (mya), while the divergence between *P. obovata* and *P. abies* began 17.6 mya. Demographic inferences retrieved the same main clusters within *P. abies* than previous studies, that is, a vast northern domain ranging from Norway to central Russia, where the species is progressively replaced by Siberian spruce (*P. obovata*) and two smaller domains, an Alpine domain and a Carpathian one, but also revealed further subdivision and gene flow among clusters. The three main domains divergence was ancient (15 mya), and all three went through a bottleneck corresponding to the LGM. Approximately 17% of *P. abies* Nordic domain migrated from *P. obovata *~103K years ago, when both species had much larger effective population sizes. Our analysis of genomewide polymorphism data thus revealed the complex demographic history of Picea genus in Western Europe and highlighted the importance of material transfer in Swedish breeding program.

## 1 INTRODUCTION

Plant and animal species in Western Europe were exposed repeatedly to ice ages and therefore went through cycles of contraction and expansion from one or multiple refugia (Depraz, Cordellier, Hausser, & Pfenninger, 2008; Petit et al., 2003; Pilot et al., 2014; Schmitt & Haubrich, 2008). The dominant paradigm of early phylogeographic studies postulated that species survived the last glacial maximum (LGM) in small refugia in Southern Europe (Hewitt, 2000; Taberlet, Fumagalli, Wust‐Saucy, & Cosson, 1998; Tzedakis, Lawson, Frogley, Hewitt, & Preece, 2002, 2003) and then recolonized Europe through different routes. However, while this still seems to be true for temperate species such as oaks (Petit et al., 2003), paleo‐ecological data (pollen fossils maps, macrofossils) as well as genetic studies indicated that boreal species were able to survive at much higher latitudes than initially foreseen. This generally led to a more diffuse and complex population genetic structure than in species with southern refugia (Lascoux, Palme, Cheddadi, & Latta, 2004; Tzedakis, Emerson, & Hewitt, 2013; Willis, Rudner, & Sumegi, 2000). In the case of genetic studies, care was generally taken to sample in natural forests, though in some cases this turned out to be difficult. For instance, in larch (*Larix decidua*) populations in central Europe, some of the discordant phylogeographic patterns corresponded to recent translocations by German immigrants of seeds from stands belonging to a different refugium (Wagner, Liepelt, Gerber, & Petit, 2015).

**Figure 1:**
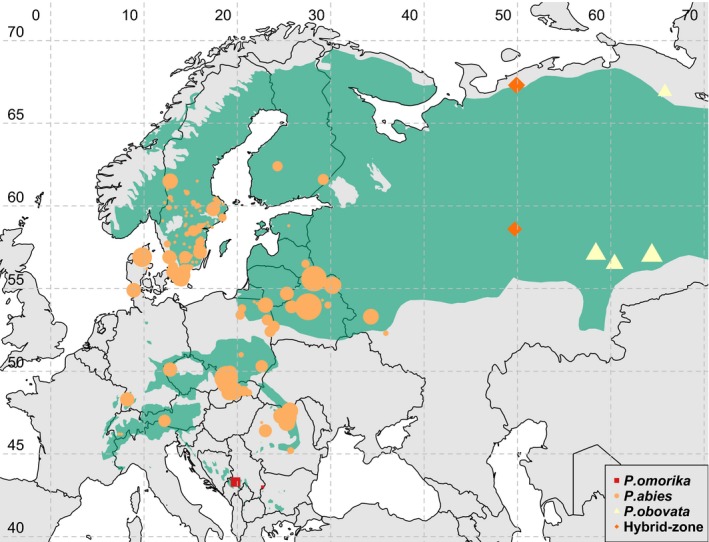
Distribution range of the three European spruce species (modified from EUFORGEN, 2009, www.euforgen.org). Points are sampling coordinates of *Picea abies* (orange disks), *Picea obovata* (yellow triangles), *Picea omorika* (red squares), the hybrid populations (gold diamonds); point sizes are proportional to the number of individuals in each location. For *P. abies*, they consist of both natural population samples and mother trees from common garden trials for the Swedish breeding program

Norway spruce (*Picea abies*) is a dominant conifer tree species in Western Europe whose current distribution is divided into three main areas: a vast northern domain ranging from Norway to central Russia, where the species is introgressed and progressively replaced by Siberian spruce (*P. obovata*), and two smaller domains, an Alpine domain and a Carpathian one (Figure 1; EUFORGEN, 2009). Earlier population genetic and phylogeographic studies based on isozymes and cytoplasmic markers, respectively, suggested that this current distribution originated from two main LGM refugia: one centered in Russia and another in the Alps (Tollefsrud et al., 2008, 2009). However, more recent studies based on nuclear sequence data suggested that northern populations were extensively admixed, unlike the southern ones that were rather homogeneous (Chen, Kallman, et al., 2012; Tsuda et al., 2016). In particular, introgression from Siberian spruce (*P. obovata*) into *P. abies* could be detected as far as Central Russia, especially in the north, resulting in a large hybrid zone centered on the Urals.

Furthermore, there is an apparent conflict in the relationship between northern populations of *P. abies*, southern ones, and Siberian spruce at nuclear (SSR) and mitochondrial markers. SSR markers define two well‐differentiated groups: southern and northern *P. abies*, on the one hand, and Siberian spruce, on the other hand (Tsuda et al., 2016). Mitochondrial DNA, in contrast, singles out southern populations of *P. abies* and grouped northern and *P. obovata* populations together (Lockwood, Aleksic, Zou, Wang, & Liu, 2013; Tsuda et al., 2016). The latter led Lockwood et al. (2013) to propose that southern populations of *P. abies* be regarded as a different species or, at least, as a subspecies. Based on the joint pattern at nuclear and mtDNA, Tsuda et al. (2016) suggested that the difference in pattern could be explained by asymmetric migration of pollen and seed. Their study also confirmed the extent of introgression from *P. obovata* into northern *P. abies* populations and linked it to the postglacial recolonization process. Two migration barriers were revealed: one in the Western Urals between the northern domain of *P. abies* and *P. obovata* and a second one between the Alps and Carpathian Mountains (Tsuda et al., 2016). The presence of three *P. abies* domains has been confirmed by studies using variation in cone morphology (Borghetti, Giannini, & Menozzi, 1988), organelle DNA markers (Achere, Favre, Besnard, & Jeandroz, 2005; Tollefsrud et al., 2008), AFLP (Achere et al., 2005), sequence data (Heuertz et al., 2006), and genomewide restriction site DNA markers (Fagernäs, 2017).

Although based on a limited number of nuclear DNA markers, previous studies also indicated extensive shared ancestral polymorphisms, suggesting a relatively recent divergence time measured on an effective population size timescale, as well as weak but significant effect of migration (Chen, Kallman, Gyllenstrand, & Lascoux, 2010; Chen, Kallman, Ma, Zaina, & Morgante, 2016; Heuertz et al., 2006; Li, Stocks, et al., 2010; Tsuda et al., 2016). Chen et al. (2016) concluded that the Fennoscandian domain split from the two southern domains of *P. abies* around 5 million years ago (mya), that is, before the Pliocene–Quaternary glaciation, which is consistent with estimates of dating based on the molecular clock (~6 mya, Lockwood et al., 2013). However, previous studies were limited to fairly simple demographic scenarios, such as “Isolation‐with‐Migration” models and, in particular, could not distinguish postspeciation contact from migration. None also considered translocations, and samples were generally assumed to be of local origin. Based on historical records on seed used in reforestation, the problem of transfer of reproductive material should be particularly acute in populations of *P. abies*, especially in southern Sweden. During the twentieth century, Sweden imported more than 210 tons of seed and more than 600 million plants, primarily for afforestation in southern Sweden. This material initially came primarily from central Europe but interest later shifted eastwards with introduction of material from Belarus, the Baltic states and Romania that proved to be more resistant to frost than central European provenances (Myking, Rusanen, Steffenrem, Kjaer, & Jansson, 2016).

The release of the *P. abies* genome and the development of reduced genome resequencing technologies (e.g., RAD‐Seq, exome capture) provide us with the opportunity to investigate genomewide pattern of diversity in hundreds or even thousands of individuals. Using whole‐genome polymorphism data, model‐based and nonparametric clustering methods allow detecting and quantifying subtle genetic admixture and migration (see Alexander, Novembre, & Lange, 2009; Galinsky et al., 2016; Pickrell & Pritchard, 2012, for examples in humans), while coalescent or diffusion‐based methods that use the joint site frequency spectrum (SFS) across multiple populations allow testing different demographic scenarios (Excoffier, Dupanloup, Huerta‐Sanchez, Sousa, & Foll, 2013; Gutenkunst, Hernandez, Williamson, & Bustamante, 2009).

In this paper, we thus investigated past demographics and recent translocations in *P. abies* using genomewide SNP data from >1,600 individuals sampled from (a) populations from southern Sweden that were used to establish the Swedish Norway spruce breeding program, (b) natural populations of *P. abies* across its natural range, and (c) two close relatives, the Siberian spruce (*P. obovata*) and the Serbian spruce (*P. omorika*).

## 2 MATERIAL AND METHODS

### 2.1 Sample collection

Samples in this study consist of 1,672 individuals of three *Picea* species (*P. abies*, *P. obovata*, and *P. omorika*) with origins extending from 43.0°N to 67.3°N in latitude and from 7.3°E to 65.8°E in longitude (Figure 1). The samples were gathered through two main channels. First, we collected needles from 1,499 individuals that were initially used to create the Swedish breeding program, that is, were selected as “plus trees” (trees of outstanding phenotype) in 20‐ to 40‐year‐old production forestry stands or selected as “superior” 3‐ to 4‐year‐old seedling genotypes in commercial nurseries. This was done across central and southern Sweden. Of those, 575 individuals had no clear records of their geographic origin; after genotypic clustering analyses, 15 of them showed high similarity to *P. omorika* and were thus treated as such in the following analyses. Second, seedlings from individuals coming from natural populations (six *P. omorika*, 53 *P. obovata*, 74 *P. abies*, and 40 hybrid individuals) were collected after seed germination in growth chambers in 2015. Those individuals were used as reference for genetic cluster definition and genotype assignation. *P. obovata* samples were collected from four Siberian populations: three along a longitudinal gradient in Southern Urals: Shalya (57.14°N, 58.42°E), Ekaterinburg (56.50°N, 60.35°E) and Tugulym (57.03°N, 64.37°E) and one in the Northern Urals: Krasnij Kamenj (66.54°N, 65.45°E). Two additional populations that are part of the *P. abies*–*P. obovata* hybrid zone were also included as follows: Indigo, from high latitude (67.27°N, 49.88°E) and Kirov, from a lower latitude (58.60°N, 49.68°E), the former having a more even contribution of the two parental species (Tsuda et al., 2016). In comparison with these two continental species, *P. omorika* has today a very tiny distribution range that is confined to mountain regions of Western Serbia and Eastern Bosnia and Herzegovina.

### 2.2 SNP identification and estimation of genetic diversity

Genomic DNA was extracted either from needles or buds in the case of the individuals from the breeding program or from needles from seedlings in the case of individuals sampled from natural populations using the DNeasy Plant Mini Kit (QIAGEN). 40,018 probes of 20 bp long were designed based on scaffolds of *P. abies* genome with RNAseq support to cover 26,219 *P. abies* gene modules (see more in Vidalis et al., 2018). A maximum of two probes per gene were observed. Library preparation and exome‐capture sequencing were performed by RAPiD Genomics, USA. Paired‐end short reads of 150 bp long were aligned to *P. abies* genome reference v1.0 (Nystedt et al., 2013) using BWA‐mem with default parameters (Li & Durbin, 2009). We extracted the uniquely aligned properly paired reads in the scaffolds/contigs, where probes were designed to bait for. PCR duplicates were removed with PICARD v1.141 (http://broadinstitute.github.io/picard), and INDEL realignment was performed by GATK (McKenna et al., 2010). HaplotypeCaller was used for individual genotype identification, and joint SNP calling was performed across all samples using GenotypeGVCFs. We then applied the same variant quality score recalibration protocols as Baison et al. (2018), which were trained on a set of ~21,000 SNPs identified from a pedigree study (Bernhardsson et al., 2018). This resulted in 2,406,289 SNPs after recalibration. SNPs were filtered following two criteria: (a) Each allele of individual genotype should be called with more than two reads and (b) more than half of the individuals should be successfully genotyped at each site. Five individuals were removed from our data due to sequencing failure. In total, 1,004,742 SNPs were retained for further analyses. Of these, 364,034 fall in the exons of 25,569 transcripts, 135,548 are synonymous, and 228,486 cause amino acid changes. Of the remaining sites, 448,698 SNPs are in introns and 192,010 fall in intergenic regions.

Genetic diversity was calculated at all sites and also at 0‐fold (coding sites at which all changes are nonsynonymous) and fourfold sites (coding sites at which all changes are synonymous, *π*_0_ and *π*_4_, respectively). Their ratio was then calculated for protein coding sequences of *P. omorika*, *P. obovata*, and the three main domains of *P. abies* (Fennoscandian, Alpine, and Carpathian). Tajima's *D* (Tajima, 1989) and pairwise population fixation indices *F*_ST_ between species and the main domains of *P. abies* (see below) were also calculated using polymorphisms in noncoding regions. The above summary statistics were calculated with custom Perl scripts (the scripts are provided in zenodo.org; https://doi.org/10.5281/zenodo.2530736; https://zenodo.org/record/2530736#.XKyJOuszbUI).

### 2.3 Population genetic clustering

Haplotype phasing was conducted using MACH v 1.0 (Li, Willer, Ding, Scheet, & Abecasis, 2010) with default parameter setting (average switch error rate 0.009); pairwise linkage disequilibrium (LD) was then calculated using the HaploXT program in the GOLD package (Abecasis & Cookson, 2000). For population clustering and admixture inference, the analyses were applied on 399,801 noncoding SNPs with significantly linked sites removed (pairwise LD ≥ 0.2 and FDR value ≤ 0.05).

EIGENSOFT v6.1.4 (Galinsky et al., 2016) was used to perform PCA on the genetic variation of *P. abies* and *P. obovota*. *P. omorika* was excluded from this PCA analysis due to its extremely high divergence from the other two species. For unsupervised population clustering, ADMIXTURE v1.3 (Alexander et al., 2009) was used with fivefold cross‐validation and 200 bootstrap replicates. The number of ancestral clusters (*K*) varied from one to eight, and the best value was chosen at the lowest value of cross‐validation error (Figure S1).

### 2.4 Geographic inference for individuals with unclear sources

Geographic origin of the 575 “Unknown” individuals for which no confident records of geographic origin were available was assessed based on their genotype similarity to ascertained individuals. *P. abies* individuals of known origin were first grouped into seven major clusters based on genetic clustering results and their origin. These individuals were used as the training dataset in a “Random Forest” regression model implemented in R software (Liaw & Wienner, 2002). The first five components of a PCA analysis were used for model fitting and to classify the “Unknown” individuals into each of the seven clusters. Fivefold cross‐validations were performed for error estimation. “Unknown” individuals were then assigned to the various genetic clusters defined from individuals from known origin. The whole regression process was repeated 1,000 times in order to estimate the confidence of each assignment.

### 2.5 Admixture inference

TreeMix v1.13 (Pickrell & Pritchard, 2012) was used to infer the direction and proportion of admixture events. A maximum‐likelihood phylogenetic tree was first built for the seven *P. abies* population clusters, *P. obovata*, and their hybrids by bootstrapping over blocks of 100 SNPs. *P. omorika* was used as an outgroup to root the tree. Admixture was tested between each pair of populations, and branches were rearranged after each significant admixture event added to the tree. The number of admixture events was estimated by minimizing the residual matrix of model compared to observed data. To avoid overfitting, we stopped adding admixture when the tree model explained over 95% of the variance. Multiple population admixtures were also examined using the *f*_3_‐test (Reich, Thangaraj, Patterson, Price, & Singh, 2009) implemented in TreeMix.

### 2.6 Demographic inference using multidimensional site frequency spectra

Effective population sizes and divergence times between the three most divergent *P. abies* clusters (i.e., the Alpine, Carpathian, and Fennoscandian) were first estimated. *P. omorika* was used to polarize the derive allele frequency, and shared polymorphic sites between *P. omorika* and target populations were excluded for stringency. In pilot runs to maximize the likelihood for individual SFS, we noted that estimates of historical *N*_e_ for all three populations are much larger than current ones suggesting that current populations had gone through bottlenecks. Therefore, a divergence model was applied with all three populations going through a sudden size contraction at *T*_Bot_. The Fennoscandian domain first split at time *T*_FAC_, followed by the split of the Alpine and Carpathian domains at *T*_AC_. To reduce model complexity, we used a constant migration rate (*m* = 1 × 10^−6^, selected based on pilot tests on *m* from [10^−8^, 1]) between all pairs of populations averaged along the whole divergence history. To examine whether migration could improve model composite likelihood, models with *m* = 0 and *m* = 1 × 10^−6^ were compared based on Akaike's weight of evidence. The aforementioned parameters were estimated by maximizing composite likelihoods based on observed 3‐dimensional joint SFS using Fastsimcoal2 v2.6.0.2 (Excoffier et al., 2013). We performed 100 iterations of parametric bootstrap to obtain 95% confidence intervals. Following (Excoffier et al., 2013) recommendation, the likelihood ratio *G*‐statistics (CLR = log_10_(CL_O_/CL_E_), where CL_O_ and CL_E_ are the observed and estimated maximum composite likelihood, respectively) was computed to evaluate model goodness of fit. A nonsignificant *p*‐value of this test indicates that the observed SFS is well explained by the model. In a second step, demographic inference was extended to all three spruce species using pairwise joint minor allele frequency spectra. For the three major domains of *P. abies*, parameter values that were estimated during the first step were used (see Figure 4 for a description of the model).

## 3 RESULTS

### 3.1 Genetic diversity and population divergence

Genetic diversity was calculated at 0‐fold and fourfold sites (*π*_0_ and *π*_4_) and their ratio calculated for protein coding sequences of *P. omorika*, *P. obovata*, and the three main domains of *P. abies* (Alpine, Carpathian, and Fennoscandian). On average, we used around 30,000 SNPs in 7,453 genes for each calculation. *P. omorika* harbored the lowest diversity (*π*_4_ = 0.0066), while *P. obovata* and *P. abies* exhibited larger values, ranging from 0.0072 to 0.0079. We found less difference in *π*_0 _values among the three species (0.0029–0.0032). This led to large *π*_0_/*π*_4 _ratios in all three species but especially in *P. omorika* (0.44 compared to 0.39 and 0.4, for *P. obovata* and *P. abies*, respectively, Table 1). Estimates of Tajimas' *D* were slightly negative but not significantly different from zero for *P. obovata* (−0.176), for *P. abies* (−0.32 to −0.42) and for their hybrid population in Kirov (−0.296). The positive value of Tajimas' *D* observed in *P. omorika* (0.875) likely reflects the sudden and very recent population contraction experienced by the species.

**Table 1:** Genetic diversity (*π* × 10^−3^), Tajima's *D*, and population divergence (*F*_ST_) estimates

	*π*_0_	*π*_4_	*π*_0_/*π*_4_	*D*	*D*_coding_	*P. omk*	*P. obv*	Kirov	FEN	ALP	CAR
*P. omk*	2.9	6.6	0.44	0.88	0.04	—	0.642	0.639	0.652	0.677	0.671
*P. obv*	3.0	7.7	0.39	−0.18	−0.05		—	0.084	0.133	0.222	0.196
Kirov	3.9	10	0.39	−0.30	−0.16			—	0.102	0.172	0.147
FEN	3.2	7.9	0.41	−0.34	−0.22				—	0.145	0.123
ALP	2.7	7.2	0.38	−0.32	−0.22					—	0.116
CAR	2.9	7.3	0.40	−0.42	−0.26						—

Abbreviations: *P. omk., P. omorika; P. obv., P. obovata. P. abies*: FEN, Fennoscandian; ALP, alpine; CAR, carpathian.

Pairwise population fixation indices (*F*_ST_) in noncoding regions were highest between *P. omorika* and *P. abies* domains (average *F*_ST_ = 0.66). The Fennoscandian domain was more closely related to *P. obovata* (*F*_ST_ = 0.13) and to the hybrid population (*F*_ST_ = 0.1) than the two other *P. abies* domains were (*F*_ST_ = 0.15–0.22, Table 1). This lends further support to the population admixture or gene flow from *P. obovata* toward *P. abies* northern populations (Tsuda et al., 2016). Within *P. abies*, genetic distances ranged from 0.12 to 0.15, Alpine and Carpathian domains being the closest, and Alpine and Fennoscandian domains the farthest (Table 1).

### 3.2 Population structure and admixture in *P. abies*

**Figure 2:**
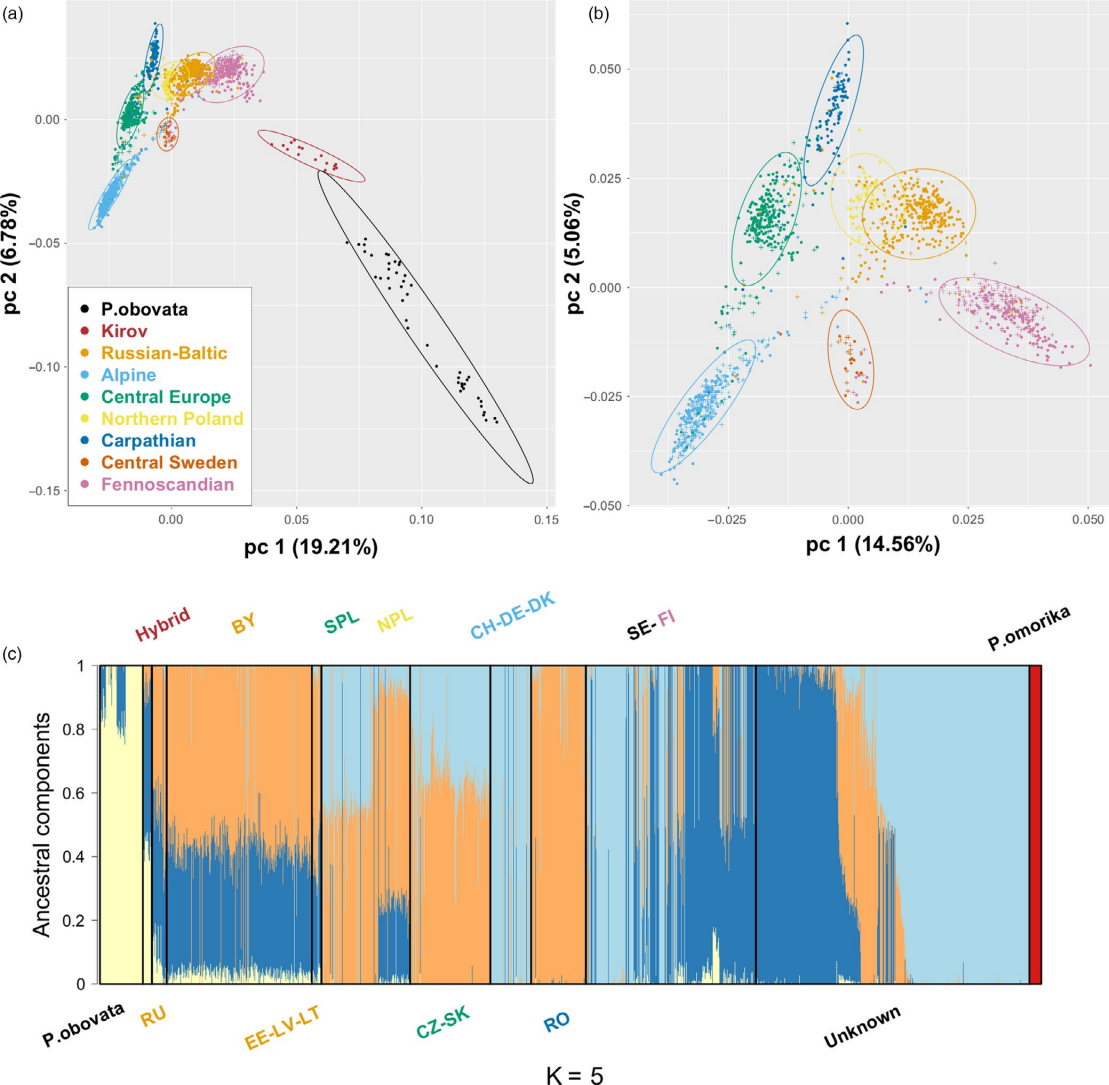
Population structure and admixture inferences of the three European spruces species. (a) Multidimensional scaling plot of PCA results (only *Picea abies* and *Picea obovata* individuals are represented), colors corresponds to different genetic clusters (see legend). The two first principal components are shown. (b) PCA results based on *P. abies* populations only. (c) Admixture analyses describing the proportion of ancestral components for *K* = 5. Red and yellow are, respectively, *Picea omorika* and *P. obovata* genetic background, while light blue, orange, and dark blue represent Alpine, Carpathian, and Fennoscandian domains of *P. abies,* respectively. Solid black lines delimit the different populations. Trees with unclear origin in records are gathered under the “Unknown” label. Text colors showed the same genetic clusters in (a) except for the Swedish clusters. Russian–Baltic: Russia (RU), Belarus (BY), Estonia (EE), Latvia (LV), Lithuania (LT); Alpine: Germany (DE), Switzerland (CH), Denmark (DK); Central Europe: Slovakia (SK), Cze‐republic (CZ), Southern Poland (SPL); Northern Poland (NPL); Carpathian: Romania (RO); Fennoscandia: Finland (FI), Sweden (SE). Note that all Swedish populations (SE) were mixed in this case

First, a principal component analysis (PCA) was conducted on genetic variation of *P. abies* and *P. obovata*. *P. omorika* was excluded from this analysis as its divergence from the two other species far exceeded the one between *P. abies* and *P. obovata* populations (Tables 1 and 3). While *P. obovata* and the hybrid populations clustered separately from *P. abies*, the range of the latter also exhibited clear population genetic structure (Figure 2a,b). Seven clusters can be distinguished within *P. abies* that are embedded within a triangle whose summits correspond to three main domains: Alpine, Carpathian, and Fennoscandian. Other clusters appear as intermediate between these three domains: Central Europe between Carpathian and Alpine, Russian–Baltics between Carpathian and Fennoscandian, and Southern and Central Sweden between the Alpine and Fennoscandian clusters. Trees from northern Poland constitute a separate cluster situated between the Russian–Baltics and the Carpathian domains.

To understand the history of the clusters detected in the PCA, we turned to unsupervised clustering analysis (ADMIXTURE, Figure 2c). Examination of the resulting Admixture plot led to the following interpretation. The three summits of the triangle in the PCA analysis, representing the Alpine, Carpathian, and Fennoscandian domains correspond to three ancestral clusters in *P. abies* populations from which other clusters are derived through admixture. For instance, the Russian–Baltics populations include components from the Carpathians and Fennoscandia, as well as a small fraction from *P. obovata*. Central European populations derived from admixture between the Alpine and Carpathian domains. Trees from Northern Poland are similar to trees from the Russian–Baltics domain but also contain an additional contribution from the Alpine domain. The Swedish populations are particularly complex: Some trees clustered with Alpine ones, others with Fennoscandia and the Russian–Baltics domain, and a small fraction correspond to admixture between the Alpine and Fennoscandian domains.

Finally, no admixture was identified in *P. omorika* but traces of a Fennoscandian contribution could be detected in *P. obovata*. The populations Indigo and Kirov were sampled from the hybrid zone at roughly the same longitude but at very different latitudes. Interestingly, only the southern population, Kirov, exhibits signs of admixture while the northern population Indigo belongs to *P. obovata* confirming that this species dominated further west at higher latitudes (Tsuda et al., 2016).

### 3.3 Prediction of geographic origin based on genotype similarity

As noted above, information on the exact origin of 575 individuals of the breeding population was missing. We therefore used the PCA coordinates of these individuals to assign them to one of the seven *P. abies* clusters established from trees sampled in natural populations. Fivefold cross‐validation showed that this method was robust (error rate <8%). Each individual was assigned to one of the seven clusters identified in *P. abies* populations by PCA, and a confidence value was computed by bootstrapping over 100 subsets of total samples. In total, 560 out of 575 (97.4%) of the individuals whose ancestry was unknown (“Unknown” individuals) could be assigned to one of the seven *P. abies* clusters with an average probability over 0.905 based on genetic similarity (Table 2), the 15 remaining belonging to *P. omorika*. Among the 560 trees that could be assigned, the two largest groups consisted of Alpine and Fennoscandian clusters (37% and 35%, respectively). The results thus suggest that the genomic markers we used in this study are powerful enough to give a high‐resolution picture of the recent divergence history in *P. abies*. Hence, the method could serve as a rather efficient way to identify translocation material used for reforestation.

**Table 2:** Inference of geographic origin of *Picea abies* trees based on genotype similarity

Cluster	Rus‐Baltics	Alpine–Nordic			Visegrad^a^	North Poland	Romania	Central‐Sweden	Fenno‐scandia
		Germany	Sweden	Denmark					
#ind.	38	27	168	13	53	28	17	21	195
Prob.	0.97	0.738	0.683	0.935	0.955	0.966	0.96	0.936	0.998

a Visegrad includes Hungary, Poland, Czech Republic, and Slovakia.

In addition, among the individuals that were sampled in common garden trials across central and southern Sweden, 290 showed a dominant Fennoscandian ancestry component, 379 displayed a dominant Alpine ancestry component that fit with postglacial recolonization paths of *P. abies* (Tollefsrud et al., 2008, 2009) and thus could have truly originated from local populations and 31 shared both ancestries. The rest exhibited various levels of contribution from the Carpathian domain or even from *P. obovata*. This suggests that at least 55% of *P. abies* trees in Central and southern Sweden are recent translocations and up to 75% when considering trees belonging to the Alpine domain as exogenous.

**Figure 3:**
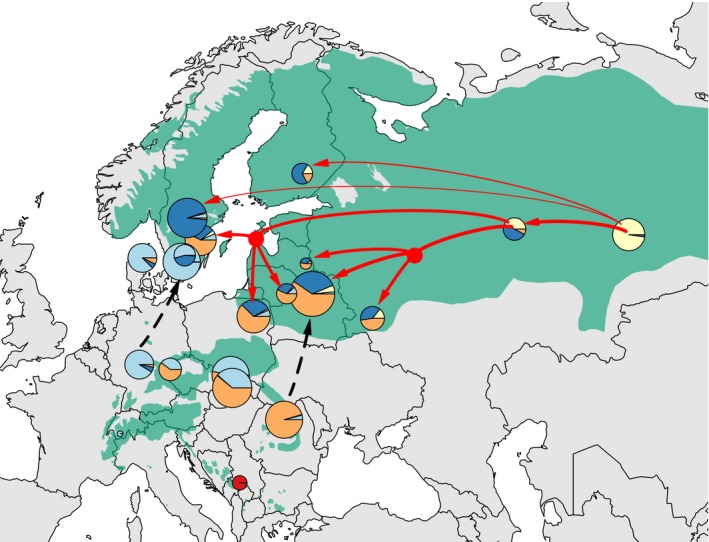
Summary of population structure and admixture in *Picea abies* from *Picea obovata*. The red arrows show the direction of the main migration events, and the line width is proportional to the migration rate. The colors in each pie refer to genetic background components estimated through unsupervised population structure (red: *Picea omorika*, yellow: *P. obovata,* light blue, orange, and dark blue: Alpine, Carpathian, and Fennoscandian domains of *P. abies,* respectively). Dark dotted lines show possible transfer and/or introgression directions from the Alpine domain to southern Sweden and from the Carpathian domain to the Russian–Baltic region

Previous analyses revealed the importance of admixture event in *P. abies* recent history in Western Europe: TreeMix v1.13 (Pickrell & Pritchard, 2012) was thus used to quantify the intensity and direction of the major admixture events between *Picea* populations (Figure S2). They were (a) from *P. obovata* to the *P. abies*–*P. obovata* hybrids (41.5% of ancestry), (b) from the hybrid to the Russian–Baltics cluster (39.0%), and (c) from *P. obovata* to the Fennoscandian cluster (12.7%) (Figure 3). All admixture events probably occurred quite recently as they were close to the tips of the tree (Figure S2). Extensive admixture among *P. abies* populations was also identified using the *f_3_*‐test, which is 3‐population generalization of *F*_ST_ that allows testing for admixture in a focal population (Reich et al., 2009). The *f_3_*‐tests were scanned from all possible pairs as parents and offspring populations using the script implemented in TreeMix. It was conducted at the population level. Both Central Europe and Fennoscandia clusters contain a few populations, and we have therefore reported an average results of *f_3_*‐statistics for these two clusters. More than half of *f_3_*‐statistics were significantly negative (56.5%) indicating admixed components in the Russian–Baltics individuals while 11.5%–22% of *f_3_*‐tests supported admixed components in Central Europe and in Fennoscandia populations (mainly from *P. obovata* and hybrid ancestry).

### 3.4 Demography inference of ancestry populations in *P. abies*

**Figure 4:**
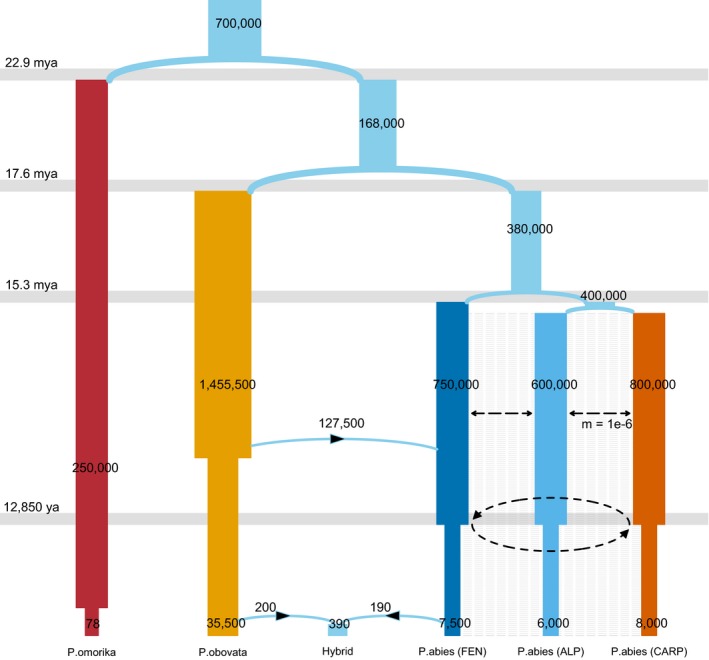
*Picea* genus demographic history in Europe. The best‐fitting model describes bottlenecks for *Picea omorika*, *Picea obovata*, and *Picea abies* (Alpine, Carpathian, and Fennoscandia domains) in different periods. The blue arrows describe the major admixture event from *P. obovata* to *P. abies* (FEN), and the hybridization between the two species. The black dotted line is migration, and the figures within bars are the effective population sizes. Migration was allowed between the three *P. abies* domains with a fixed rate of 1 × 10^‐6^. The parameters were estimated with FastSimCoal2

Multidimensional SFS was first used to estimate the demographic history of the three most divergent *P. abies* clusters, Alpine (ALP), Carpathian (CAR), and Fennoscandian (FEN), which also represent the three main ancestry components of *P. abies* in the previous analyses. Assuming an average migration rate of 10^−6^ leads to similar estimates of *N*_e_ for the three *P. abies* domains, around 6,000 to 8,000 (95% CI: 5,000–11,400), with CAR and FEN populations slightly larger than ALP (Table 3 and Figure 4). The ancestral effective population size of *P. abies* was much larger with an estimated value around 2.5 × 10^5^ to 5.7 × 10^5^. Assuming a generation time of 25 years and a mutation rate of 1.1 × 10^−9^ per site per year (Chen, Uebbing, et al., 2012; Nystedt et al., 2013; Willyard, Syring, Gernandt, Liston, & Cronn, 2007), the three populations had a similar divergence history since 15 million years ago (95% CI: 9–17.7 mya). More recently, the populations went through a bottleneck around 13,000 years ago (95% CI: 6,400–33,000), which approximately corresponds to the end of LGM.

**Table 3:** Demographic parameter estimates for *Picea omorika (OMO)*, *Picea obovata* (*OBO*), *Picea abies* main domains and *P. abies*–*P. obovata* hybrids (*HYB*)

Parameters	Point estimation	95% CI
Effective sizes		
*N_OMORIKA_*	78	42–566
*N_OBOVATA_*	35,498	14,500–57,200
*N_hybrid_*	390	120–34,500
*N_ALPINE_*	5,982	5,060–9,100
*N_CARPATHIAN_*	8,043	6,200–11,400
*N_FENNOSCANDIAN_*	7,540	6,400–9,500
Divergence times		
*T_OMO_OBO_ABIES_*	22,875,400	22,770,000–52,700,000
*T_OBO‐ABIES_*	17,600,050	17,040,000–21,493,000
*T_OBO‐HYB_*	17,597,625	17,536,000–20,281,300
*T_FAC_* a	15,274,375	9,410,000–17,673,500
*T_AC_* b	15,272,700	9,394,400–17,650,000
Admixture times		
*T_ADM_OBO‐HYB_*	103,150	1,300–242,000
*T_ADM_OBO‐ABIES_*	1,600	420–77,000
Bottleneck times		
*T_BOT_ABIES_*	12,850	6,400–32,700
*T_BOT_OMORIKA_*	2,775	900–27,200

a Fennoscandian split from Alpine and Carpathian.

b Alpine—Carpathian split.

Average migration rates (m) were set to 10^−6^ between the three main *P. abies* clusters. Given population sizes before bottleneck, it corresponded to approximately three migration events per generation per cluster. To assess if considering *m* > 0 improved the model likelihood significantly, composite likelihood parameter estimation was repeated with a null migration rate, *m* = 0. The Akaike's weight of evidence supported a model with *m* > 0 in 95% of the runs. Demographic parameters were also inferred with free varying migration rate: The median migration rate was about 5.9 × 10^−6^. However, Akaike's weight of evidence supported models considering a fixed migration parameter in 76 out of the 100 runs despite a slightly lower composite likelihood ratio (CLR) of models with migration free to vary.

The model goodness of fit was estimated by computing the likelihood ratio *G*‐statistic test. For multidimensional model fitting, the *p*‐value is highly significant which suggests that our model is certainly oversimplified and does not capture the full history of the species. However, likelihood ratio test based on a joint 2‐dimensional SFS supports our divergence model (Figure S3), indicating that, in spite of its simplified nature, it does capture the major features of the SFS (Anscombe residuals for pairwise joint SFS fitting are presented in Appendix S1).

### 3.5 Divergence history of the three spruce species

We further extended our demographic inferences to all three spruce species and the hybrid between *P. abies* and *P. obovata* based on pairwise joint SFS. *P. omorika* has a particularly small effective population size (N0_omo < 100), which is expected as the species is endangered and is part of the IUCN Red List. The natural range of *P. omorika* is believed to have been under continuing contraction since the LGM, but our estimates suggested a severe and recent bottleneck (~3,200‐fold) around 2,800 years ago. *P. obovata* has the largest Ne of the three species, with a value ~ 35,500. Unlike the other two species, no recent size contraction was detected in *P. obovata* after the end of LGM. The split of *P. omorika* occurred 23 mya, while the divergence between *P. obovata* and *P. abies* began 17.6 mya. Approximately 17% of *P. abies* FEN population migrated from *P. obovata* 103,000 years ago, when both species had much larger effective population sizes (6 × 10^5^ for FEN and 5.6 × 10^6^ for *P. obovata*). The hybrid population has a small effective population size around 400 and was established ~1,600 years ago, with almost equal contribution from both species (Figure 4). The goodness of fit (GOF) for the model can be found in Appendix S2. We also provided rescaled results to account for uncertainty about generation time (Table S1). With a 50‐year generation time, the estimates of effective populations size got halved but the divergence time in year remained the same.

## 4 DISCUSSION

In the present study, we showed that the current distribution of genetic diversity in *P. abies* is the result of a complex mixture of ancient and recent demographic events and cannot be explained by relying on a simple phylogeographic paradigm. This history involves ancient splits, repeated hybridization events, severe bottlenecks, population movements, and, very recently, important transfer of forest reproductive material associated with 19th‐ and 20th‐century afforestation efforts. Below, we discuss the most salient features of our demographic inferences, starting with their impact on genetic diversity at synonymous and nonsynonymous sites.

### 4.1 Sources of sequencing errors

There are two major possible sources of errors that could influence our inference on population admixture and demographics. First, our dataset was derived from coding regions or their flanking regions, whose allele frequencies could be affected by natural selection and thus would not be adequate for demographic inference. By calculating *π*_0_/*π*_4_ ratios at coding sequences, Chen, Glemin, and Lascoux (2017) showed that the proportion of mutations that are putatively under weak purifying selection is non‐negligible, especially for conifer trees. In the present study, *π*_0_/*π*_4_ ratio is ~0.4 but Tajima's *D* values tend to be small suggesting that purifying selection did not strongly affected the site frequency spectrum. Furthermore, linkage disequilibrium in spruce decays very fast within genes (within ~200 bp) (Chen, Kallman, et al., 2012; Chen et al., 2014; Heuertz et al., 2006) so linked positive selection is also not likely to have affected nearby SNPs through hitchhiking or selective sweeps.

A second possible source of error comes from the fact that *P. abies* genome is incomplete and highly fragmented, which increases the risk of detecting false‐positive SNPs due to paralog sequences. To avoid this issue, we first aligned reads to the whole spruce genome instead of the baited sequences directly and chose for proper paired reads aligned to one unique position in the whole genome. Mapping to paralog sequences could also distort the distributions of quality scores. Instead of hard filtering based on arbitrary cutoffs, we applied a protocol of variant quality score recalibration, parameters of which were trained using set of SNPs discovered in a pedigree study (Baison et al., 2018; Bernhardsson et al., 2018). This should help reduce false‐positive discovery but may also bias toward shared SNPs and less private ones in *P. obovata* and *P. omorika*. It could result in overestimates of admixture or migration rates between *P. obovata* and *P. abies* and therefore underestimates the divergence time among three species. However, the influence here is most likely minor since admixture is mainly in the hybrid and East European regions, which agrees with the conclusions of Tsuda et al. (2016) that was based on 10 SSR loci but with a much more extensive sampling across the ranges of *P. abies* and *P. obovata*. Furthermore, the distributions of variant quality scores for our SNP dataset also provided strong supports for our quality controls (Figure S4).

### 4.2 Genetic diversity

The *π*_0_/*π*_4_ is generally interpreted as a measure of the efficacy of purifying selection. However, it is also influenced by demography and, in particular, high values of *π*_0_/*π*_4 _can also result from the fact that the nonsynonymous diversity (*π*_0_) reaches equilibrium faster than synonymous diversity (*π*_4_), after a bottleneck (Brandvain & Wright, 2016; Gordo & Dionisio, 2015; Gravel, 2016). In the present case, the elevated *π*_0_/*π*_4_ ratio in *P. omorika* is mainly due to a decrease in *π*_4_ in that species (0.0066 compared to 0.0075 on average), due to the strong bottleneck experienced by the species as confirmed from our demographic simulations. Interestingly, a similar decrease in genetic diversity was observed by Kuittinen, Muona, Karkkainen, and Borzan (1991) when they compared variation at 19 allozyme loci in *P. omorika* and *P. abies* (expected heterozygosity *H*_e_ = 0.13–0.15 in *P. omorika* versus 0.22 in *P. abies*). The estimates of synonymous diversity in the other two species were close to the value reported in interior spruce (0.0073) by Hodgins, Yeaman, Nurkowski, Rieseberg, and Aitken (2016), however, with much smaller nonsynonymous diversity (0.0013) in the latter. In the case of interior spruce, the choice of conserved ortholog genes with lodgepole pine (*Pinus contorta*) may have led to strongly underestimate the genetic diversity at nonsynonymous sites compared to synonymous sites. In general, the *π*_0_/*π*_4 _ratio of spruce species is among the highest among perennial plants though it is still lower than in *Quercus robur* (~0.5) and other oak species (T. Leroy, pers. comm.) (Plomion et al., 2018). Possible explanations for the high *π*_0_/*π*_4 _ratio could be a more long‐lasting effect of bottlenecks in long‐lived organisms and/or a higher mutation rate per generation in trees than in annual plants. The latter could lead to accumulation of deleterious somatic mutations that can be inherited by the next generation.

### 4.3 Admixture and extensive translocation from central Europe to southern Sweden

As in earlier studies, we identified three major genetic clusters (Alpine, Carpathian, and Fennoscandian) in *P. abies* and extensive admixture from *P. obovata* (Achere et al., 2005; Borghetti et al., 1988; Bucci & Vendramin, 2000; Collignon, Sype, & Favre, 2002; Fagernäs, 2017; Heuertz et al., 2006; Tollefsrud et al., 2008; Tsuda et al., 2016). However, power provided by the large number of polymorphisms yielded a much more detailed and complex picture of the relationship among the *P. abies* main genetic clusters. In particular, admixture between the different clusters is much more frequent than suggested by previous studies, especially between the Alpine and Carpathian regions, and across the Baltic–Scandinavian regions. While the main thrust of the recolonization of previously glaciated areas after the last LGM, as well as previous waves of recolonization, stemmed primarily from the East there were also south–north movements as shown by a recent study of the vegetation in Poland during the Eemian Interglacial (130,000–115,000) and the periods that followed (Kupryjanowicz et al., 2018). During the Eemian Interglacial, Kupryjanowicz et al. (2018) inferred a recolonization from the northwest and during the Holocene a recolonization of Poland from both the northwest and the southeast.

### 4.4 Identifying recent transfers of material

The presence in central and southern Sweden, as well as Denmark, of trees with ancestry tracing back entirely, or almost entirely, to the Alpine or the Carpathian domain, reflects recent introductions. That these trees are recent introduction is supported by the fact that those individuals could be assigned to the same clade of Baltic and central European populations with very high confidence in both PCA and TreeMix analyses. Most likely, these genotypes with an important Alpine or Carpathian component reflect translocations from central European countries that took place during the massive reforestation of the twentieth century. Indeed, since the 1950s, Sweden and Norway, and to a lesser extent Finland, started to import seeds for forest reproduction material from the Belarus, Czech Republic, Germany, Slovakia, and the Baltic States (Myking et al., 2016). This is also documented in the Forestry Research Institute of Sweden (Skogforsk) records that indicate that 46 trees originated from German seedlots. Hence, our study illustrates that genomic markers could serve as an efficient way to identify and track the origins of translocations back to their origins. In addition, this study provides a prerequisite for investigating the genetic adaptive effects of translocation, which is of great importance for tree breeding, conservation, and forest management under climate change.

### 4.5 Difference in demographic histories across spruce species

In contrast to other spruce species that had a negative Tajima's *D*, *P. omorika*'s Tajima's *D* was positive and relatively high, supporting the presence of a recent and sudden contraction of its population size. The most plausible demographic scenario for *P. omorika* indeed indicates a 3,000‐fold reduction in population size around 2,800 years ago. This corresponds to the onset of the Iron Age and could have been followed by a severe depletion of forest resources although paleobotanical data from southwestern Bulgaria suggest that the onset of the Iron Age was associated with a reduction of *Abies alba* and *Pinus* sp. but a concomitant increase of *Fagus sylvatica* and *Picea abies* (Marinova, Tonkov, Bozilova, & Vajsov, 2012). The species harbored before that time a rather stable and large population size, of the same order of magnitude to that of *P. abies*. The estimate of current effective population size is extremely small for *P. omorika*, which is consistent with the fact that a limited number of trees of *P. omorika* are distributed in small stands across Western Serbia and Eastern Bosnia and Herzegovina (Aleksić, 2017), whereas the other two spruce species are continentally distributed. More recently, a possible cause for the rapid decline of Serbian spruce could be a physiological stress induced by global warming that makes *P. omorika* more susceptible to *Armillaria ostoyae*. This pathogen is reported as a major cause for root rot and drying of the crown and the infected trees die within five to six years (Aleksić et al., 2017; Ivetić & Aleksić, 2016). More generally, *P. omorika* could suffer from high temperatures. Gigov (1956) analyzed pollen abundance in peat profiles at five locations across Serbia and found that during the Subboreal period (5–2.5 ka BP), when the climate was continental, dry and warm, the percentage of *Picea cf. excelza* pollen declined at all studied localities, even at lower altitudes at which it was present during the previous Atlantic period (9–5 ka BP).

In contrast to the drastic change experienced by *P. omorika*, the three major domains of *P. abies* had similar effective population sizes, with the Alpine domain only slightly smaller. The three domains almost split at the same time and thereafter remained rather stable until the end of the GLM. *P. obovata* had a much larger population size and the bottleneck occurred earlier, around the end of the Eemian interglacial to the beginning of the last glacial. These results possibly reflect the fact that spruce species, as well as other tree species, especially in Siberia, actually occupied a much larger area before and during the LGM than assumed under the “southern refugia” paradigm that has dominated phylogeography until recently (Lascoux et al., 2004 and reference therein). The new picture that is emerging is one under which nonglaciated areas consisted in a tundra with scattered tree stands as found today at the tree line rather than an herbaceous tundra with trees only present in well‐delineated southern refugia. Trees might even have been able to survive in small pockets in glaciated areas (Carcaillet, Latil, Abou, Ali, & Ghaleb, 2018; Quinzin, Normand, Dellicour, Svenning, & Mardulyn, 2017; Tzedakis et al., 2013).

Regarding divergence time, our result pushes the split of the three major domains in *P. abies* further back in time (15 mya), much older than the 5–6 mya from Lockwood et al. (2013); and Chen et al. (2016). A major reason could be that both latter estimates were based on admixed samples in either so‐called “northern” or “southern” populations. The relatively recent admixture events between the three major domains could significantly reduce these estimates. While admixture could explain why previous estimates could be underestimates, the new divergence times nonetheless seem at variance with a median node age of less than 5 mya obtained for northern clades by Leslie, Beaulieu, Rai, Crane, and Donoghue (2012) although it should be pointed out that these estimates, in contrast, may as well err on the low side. For example in pines, a new fossil calibration led to an estimate of the origin of crown Pinus that is likely up to 30 mya older (Early Cretaceous) than inferred in most previous studies (Late Cretaceous) (Saladin et al., 2017). We also estimated the split of *P. abies* and *P. obovata* to be around 18 mya and the split with *P. omorika* to be around 23 mya. This latter estimate is consistent with a divergence of the Eurasian spruce clade around the early to the middle Miocene (13 ~ 23 mya), as inferred from molecular clock dating methods (Leslie et al., 2012; Lockwood et al., 2013).

## 5 CONCLUSION

Two main conclusions emerge from the present work. First, the current distribution of *P. abies* is the result of complex population movements and admixture events with at least three major ancestral groups (Alpine, Carpathian, and Fennoscandia) and derived ones. As in Tsuda et al. (2016), we observed that admixture of *P. obovata* extended as far west as Fennoscandia and the Baltics.

Second, a large proportion of the trees selected in southern Sweden to establish the Swedish breeding program were shown here to be recent introductions from Central Europe. This agrees well with previous studies of the release of “alien” material in Sweden (Laikre, Palme, Josefsson, Utter, & Ryman, 2006; Myking et al., 2016) that reported a large use of material of foreign origin in Swedish forestry. The trees genotyped in the present study were “plus trees” used to establish the breeding program and were therefore selected based on their superior phenotypes. Hence, it already suggests that the use of alien material did not have any strong negative effect on growth and productivity. In addition, since these trees have been tested through a large number of progeny or clonal tests across southern Sweden, this material provides a unique opportunity to test for local adaptation in *P. abies* and this is the object of a companion paper (Milesi et al., 2018).

## CONFLICT OF INTEREST

None declared.

## Data Availability

Raw reads are available in SRA NCBI database (https://www.ncbi.nlm.nih.gov/sra/) deposited as PRJNA511374 bioproject. Scripts and datasets are deposited in Zenodo database (https://zenodo.org/) under https://doi.org/10.5281/zenodo.2530736 (Chen et al., 2019).
