# Supplementary figures and images for: Genomic data provide new insights on the demographic history and the extent of recent material transfers in Norway spruce
Source: Evol Appl. 2019 Apr 30;12(8):1539–51. doi: 10.1111/eva.12801 (PMC6708423; doi:10.1111/eva.12801)

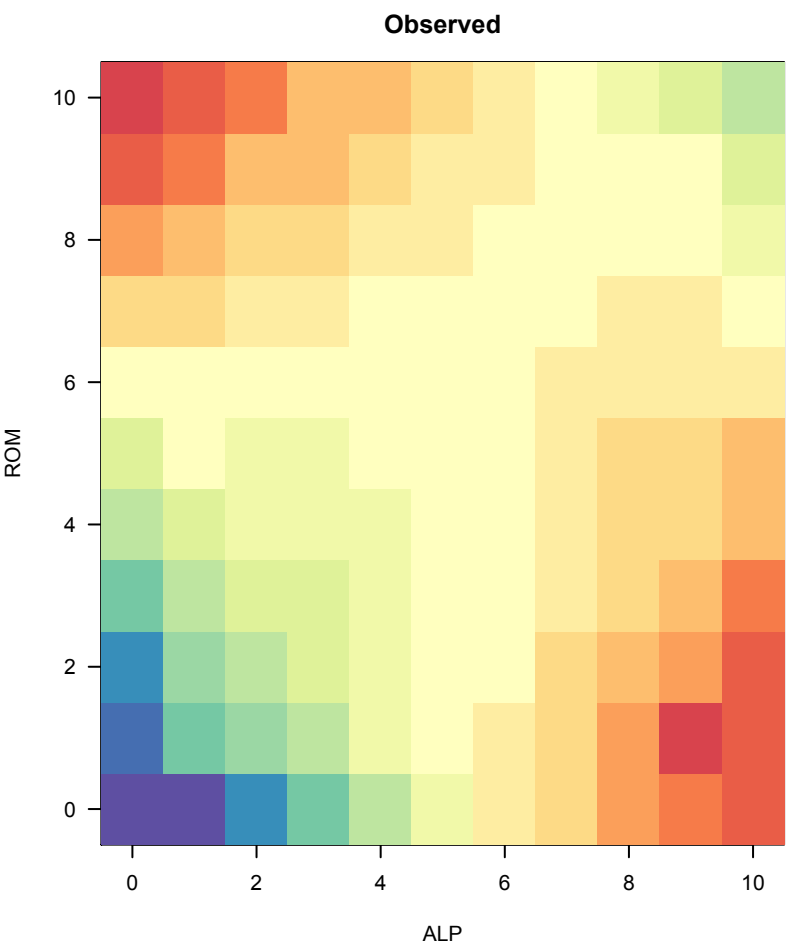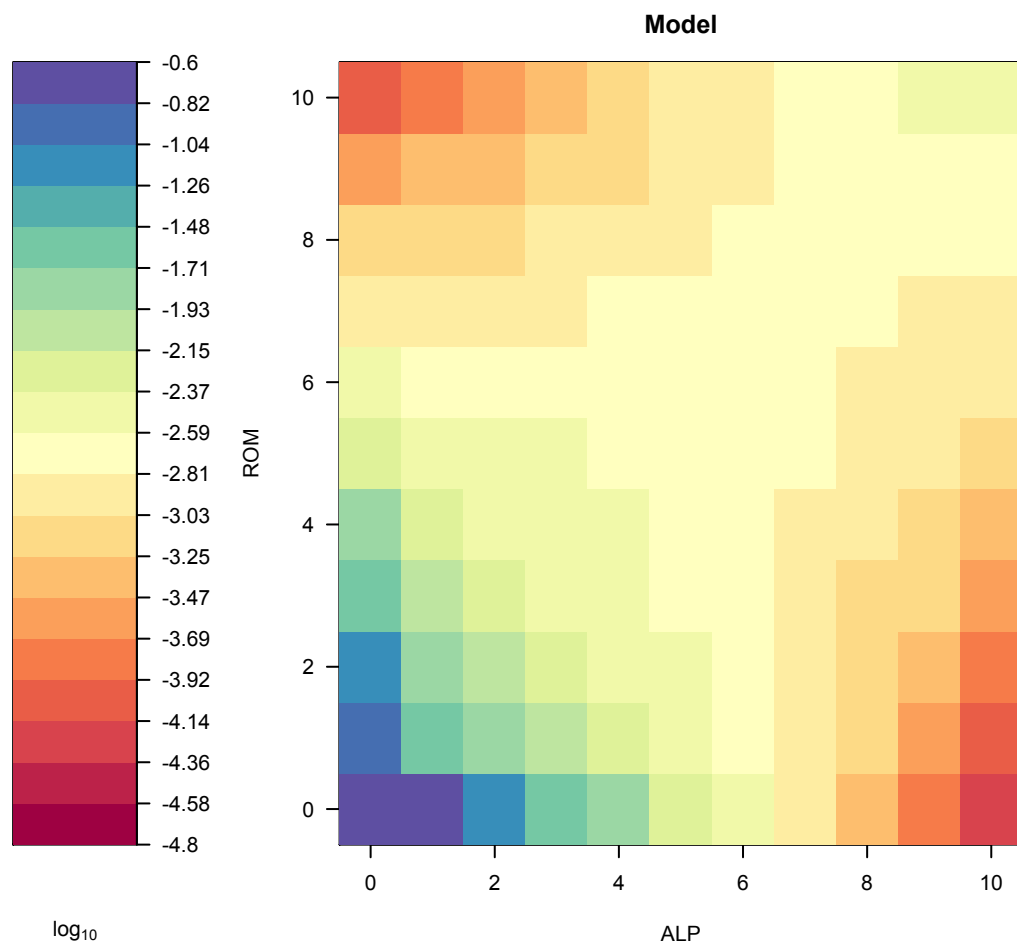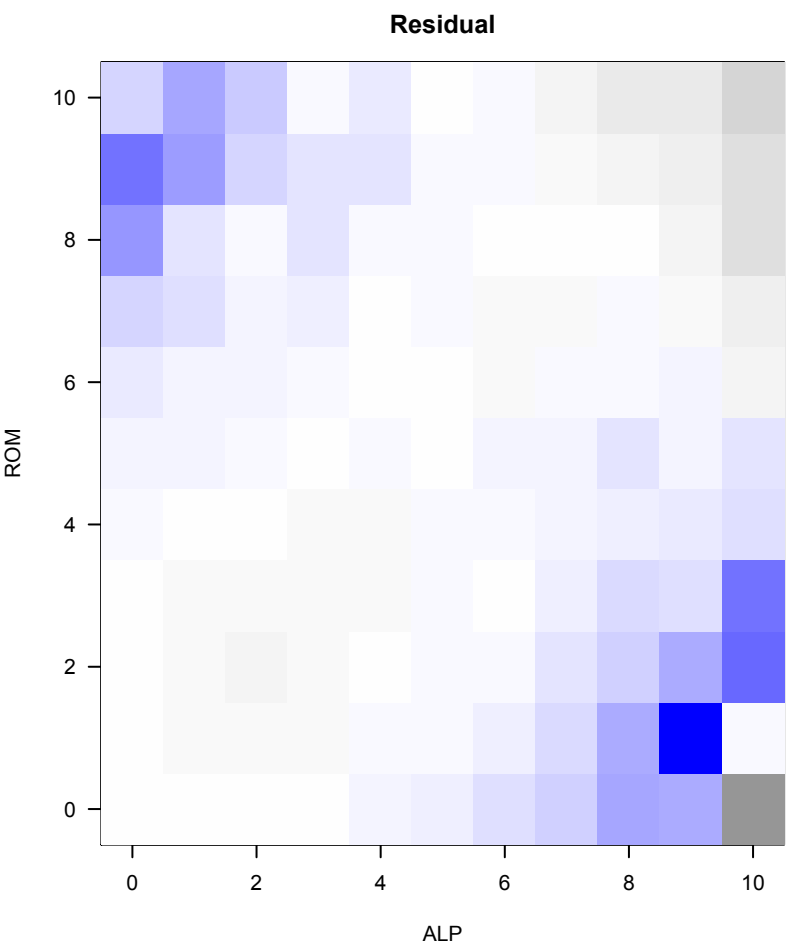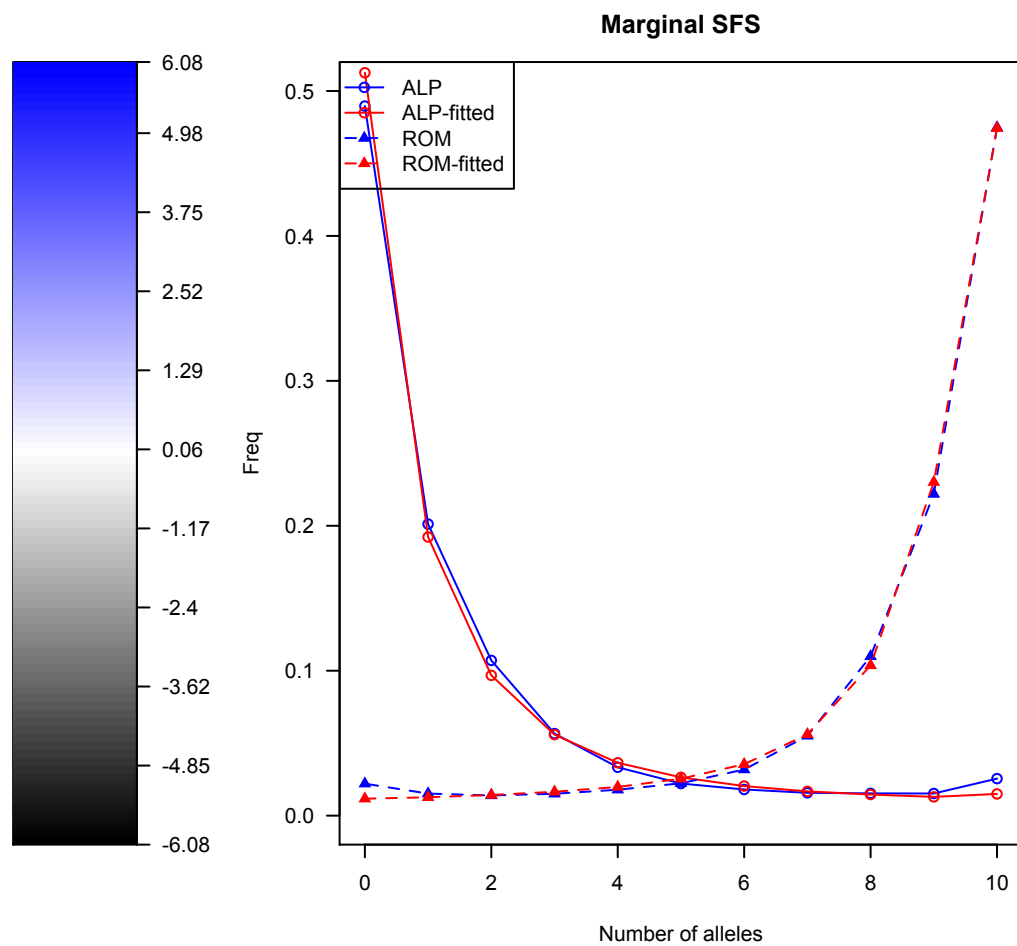

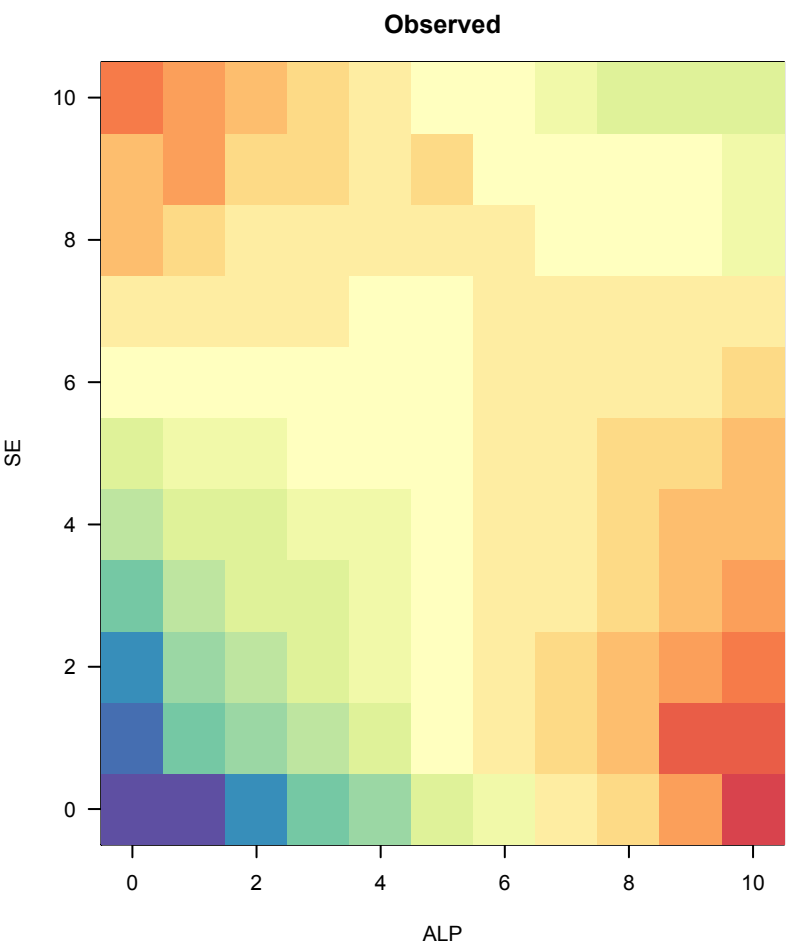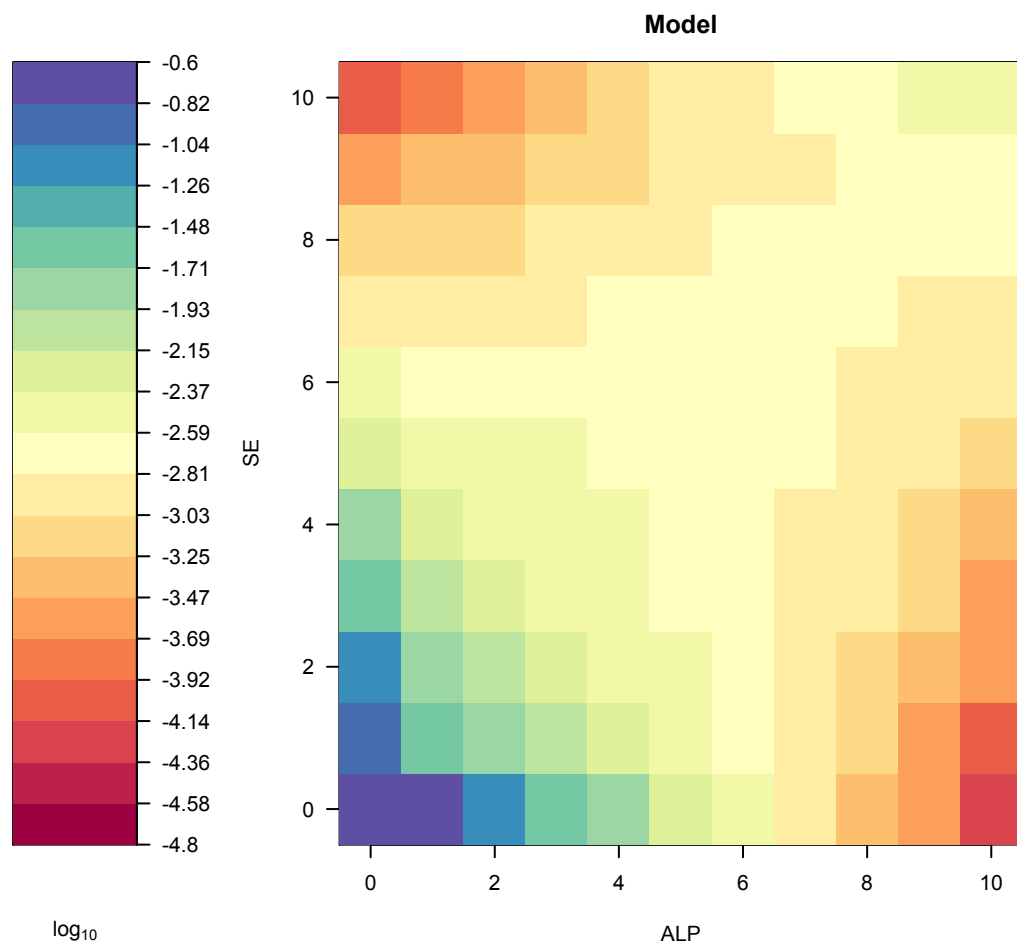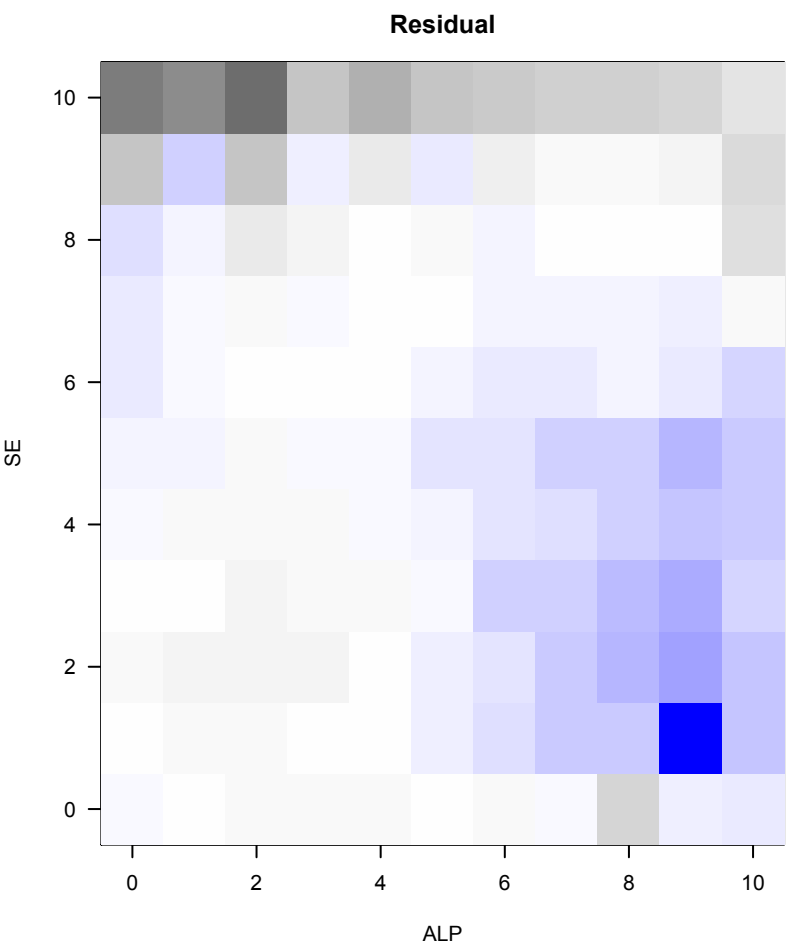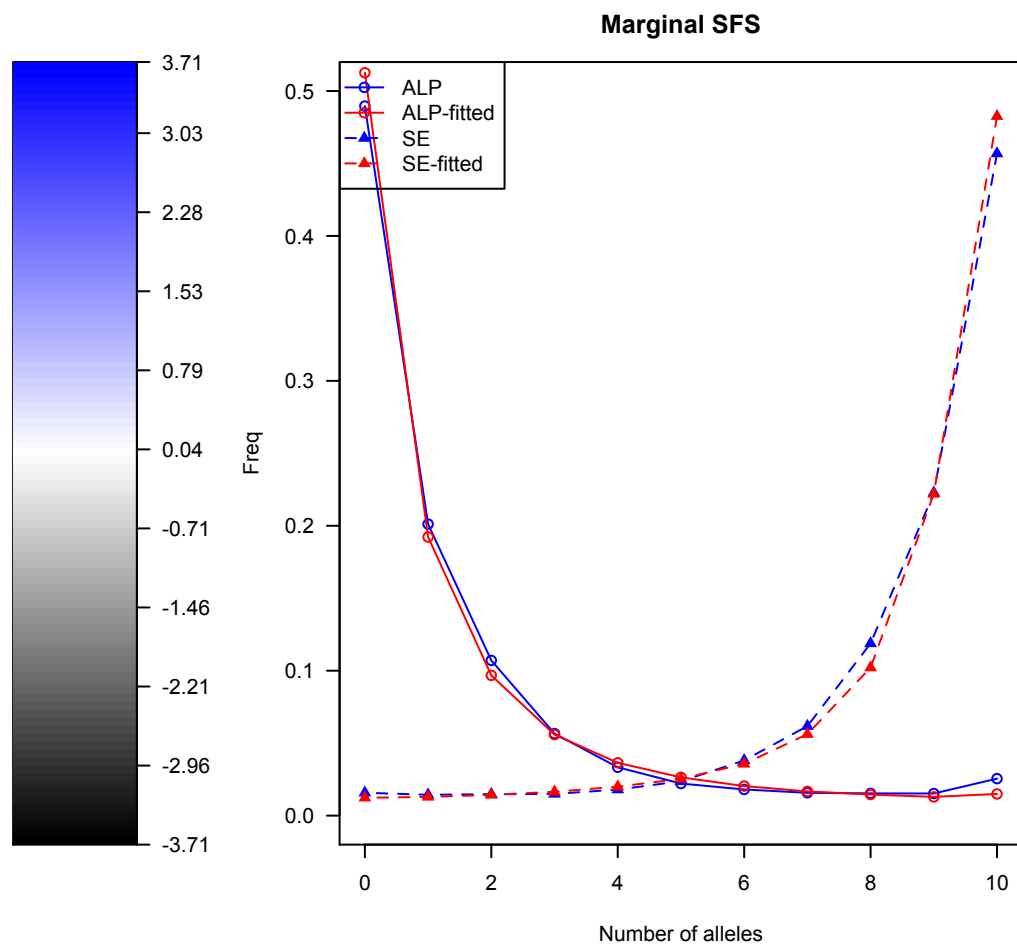

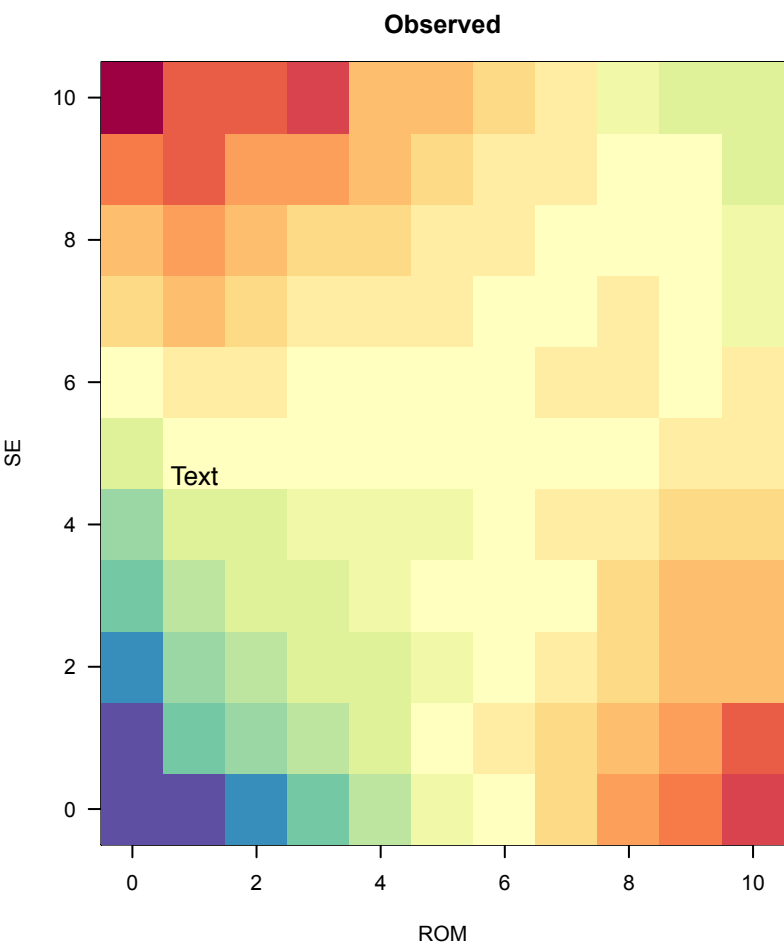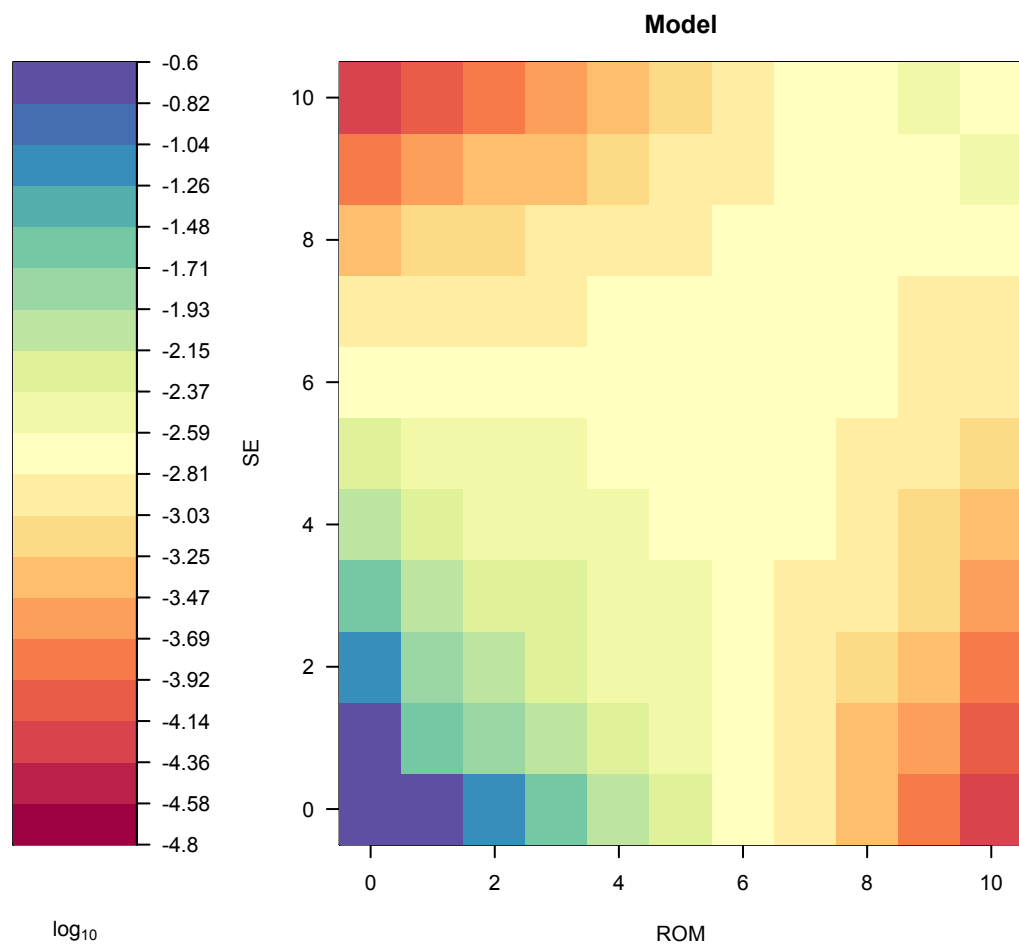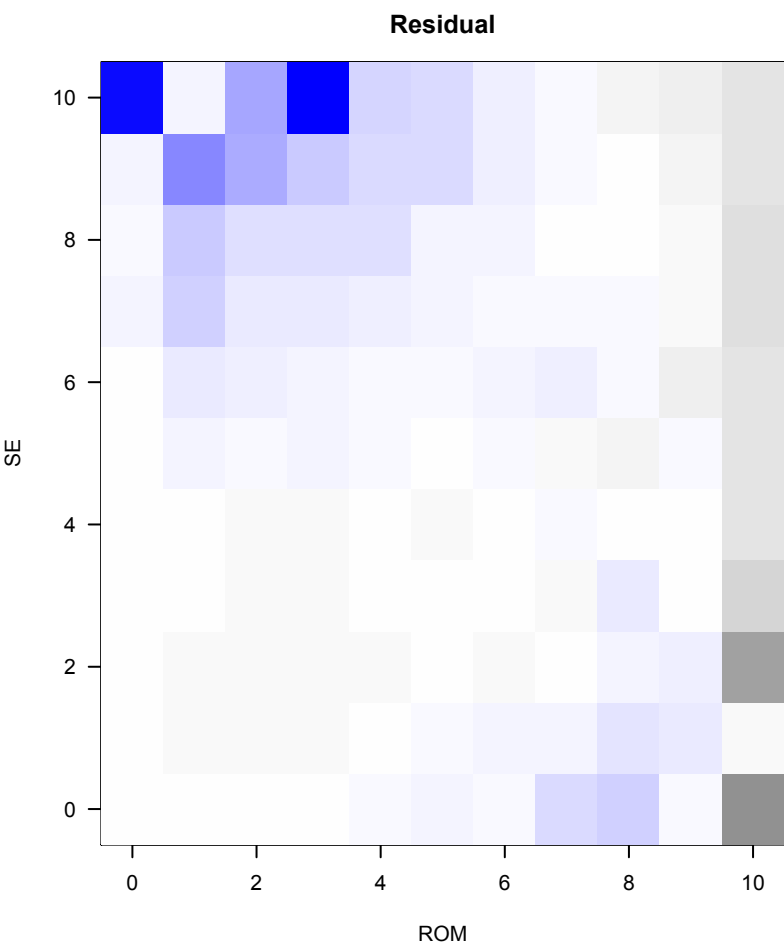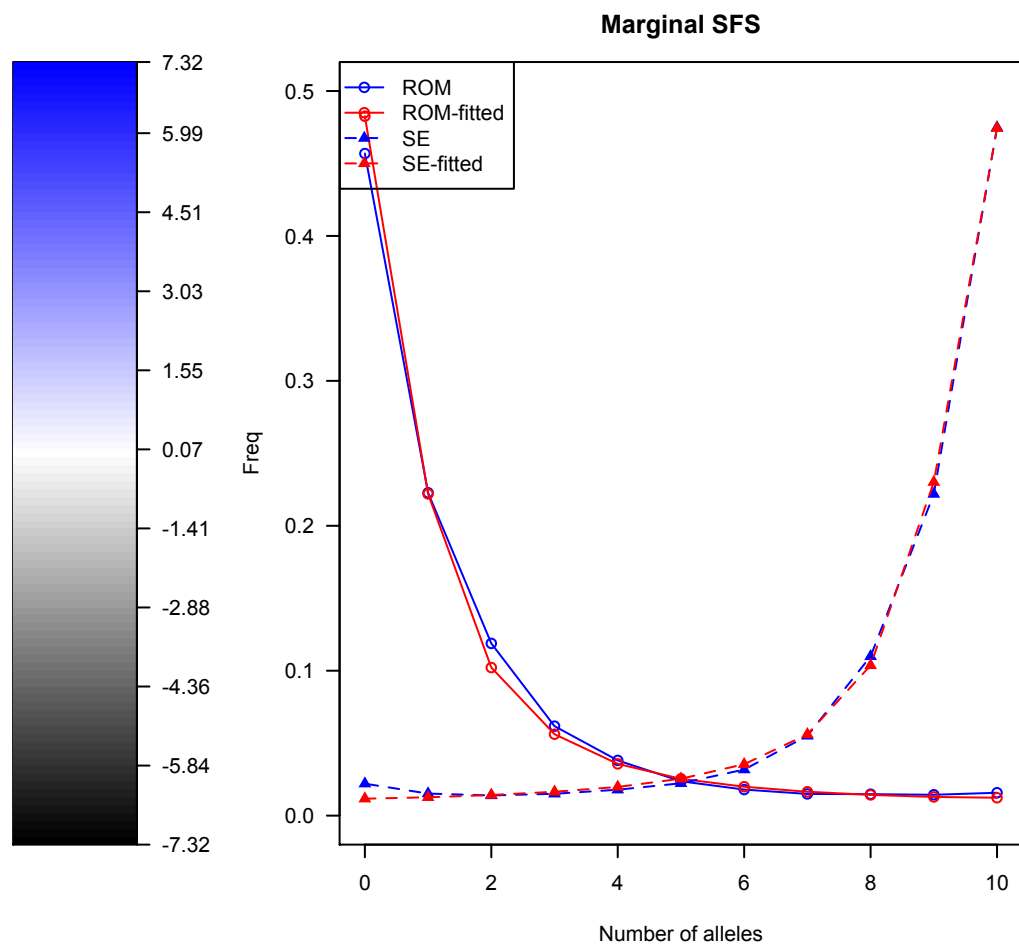

Supplement: Supplementary file 1 [file EVA-12-1539-s001.pdf]
