# Supplementary material for: Genomic data provide new insights on the demographic history and the extent of recent material transfers in Norway spruce
Source: Evol Appl. 2019 Apr 30;12(8):1539–51. doi: 10.1111/eva.12801 (PMC6708423; doi:10.1111/eva.12801)
Supplement: Supplementary file 3 [file EVA-12-1539-s003.pdf]

Genomic data provides new insights on the demographic history and the extent of recent material transfers in Norway spruce

Supporting Information

Table S1: Demographic parameter estimates rescaled by a generation time of 50 years for *P. omorika (OMO)*, *P. obovata* (*OBO*), *P. abies* main domains and *P. abies* – *P. obovata* hybrids (*HYB*).

Parameters	Point estimation
$N_{OMORIKA}$	40
$N_{OBOVATA}$	17,749
N_{HYBRID}	200
N_{ALPINE}	2,991
$N_{CARPATHIAN}$	4,022
$N_{FENNOSCANDIAN}$	3,770
$T_{OMO\_OBO\_ABIES}$	22,875,400
$T_{OBO-ABIES}$	17,600,050
$T_{OBO-HYB}$	17,597,625
T_{FAC}	15,274,375
T_{AC}	15,272,700
$TADM_{\_OBO-HYB}$	103,150
$TADM_{\_OBO-ABIES}$	1,600
$TBOT_{\_ABIES}$	12,850
$TBOT_{\_OMORIKA}$	2,775

[a] Fennoscandian split from Alpine and Carpathian
[b] Alpine – Carpathian split

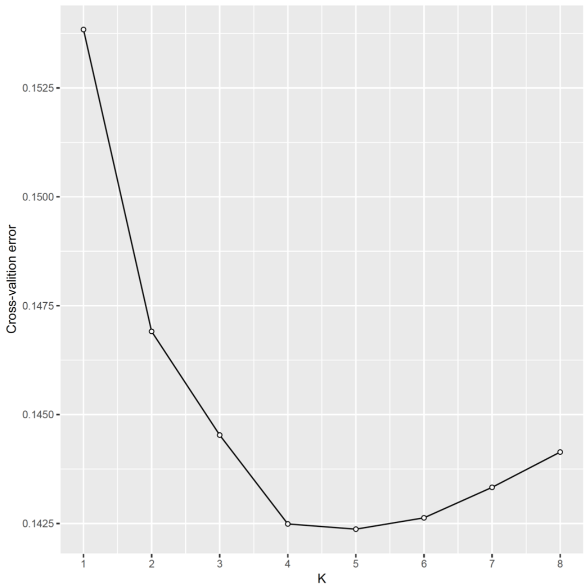

Figure S1: Cross-validation error regarding number of cluster for unsupervised population clustering.

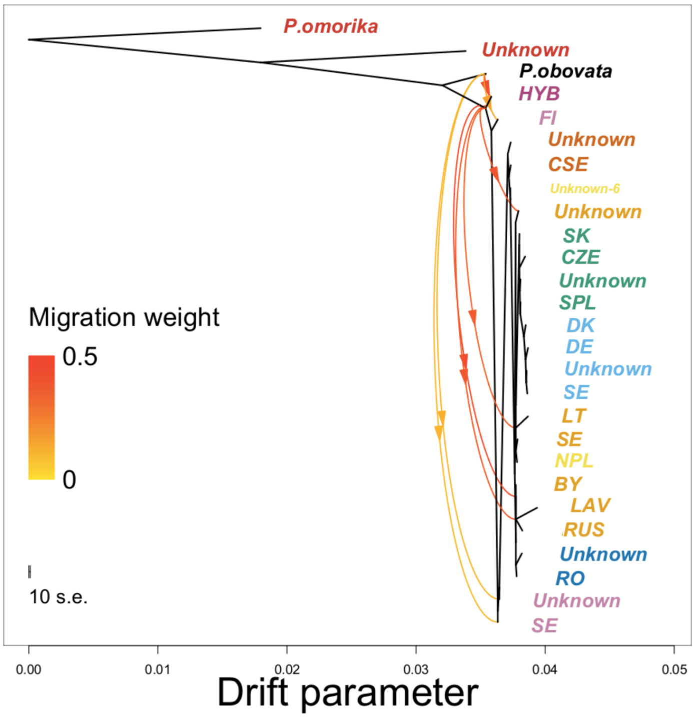

Figure S2: TreeMix graph with 8 migration events. Text colors showed the same genetic clusters in (Figure 2a and b). Russian-Baltic: Russia (RU), Belarus (BY), Estonia (EE), Latvia (LV), Lithuania (LT); Alpine: Germany (DE), Switzerland (CH), Denmark (DK), Sweden (SE); Central Europe: Slovakia (SK), Cze-republic (CZ), Southern Poland (SPL); Northern Poland (NPL); Romania (RO); Central Sweden (CSE); Fennoscandia: Finland (FI), Sweden (SE).

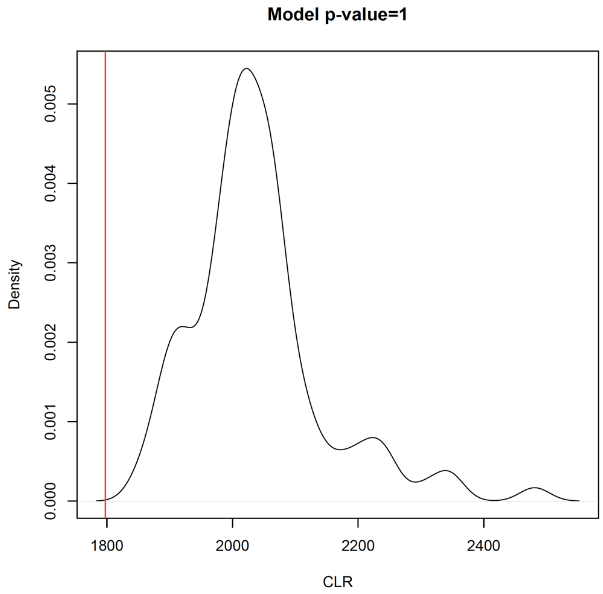

Figure S3: Likelihood ratio G-statistics distribution. The likelihood ratio G-statistics ($CLR = \log_{10}(CL_O/CL_E)$, where CL_O and CL_E are the observed and estimated maximum composite likelihood, respectively) was computed to evaluate model goodness-of-fit. A non-significant *p*-value of this test indicates that the observed SFS is well explained by the model. The red dotted line is the CLR of our divergence model.

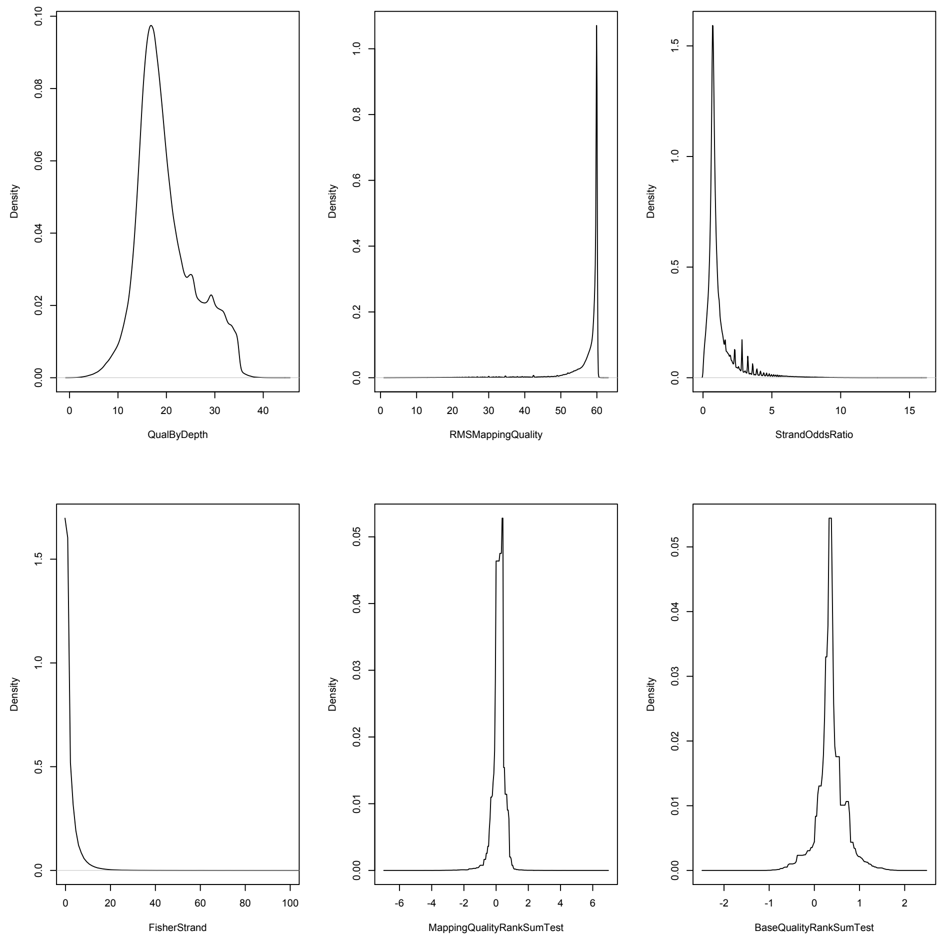

Figure. S4 Variant quality scores reported for final SNP dataset after VQSR using GATK toolkit. Density distributions of six quality scores (QD, MQ, SOR, FS, MQRankSum, and BaseQRankSum) were plotted to compare with generic recommendations (QD<2; MQ<40; FS>60; SOR > 3; MQRankSum < -12.5) for hard-filtering provided by Broad Institute.
